# The optimization method of wing plasma ice shape regulation based on quantitative assessment of flight risk

**DOI:** 10.1038/s41598-024-61049-8

**Published:** 2024-05-06

**Authors:** Zhe Li, Pengfei Dou, Qiao Huang

**Affiliations:** Aviation Engineering School, Air Force Engineering University, Xi’an, 710038 China

**Keywords:** Quantitative assessment of flight risk, Flight safety, Ice shape regulation, Plasma, Aerospace engineering, Engineering

## Abstract

Plasma ice shape regulation is a technology which uses plasma actuator to regulate the continuous ice into safer intermittent ice by its significant thermal effect with limited energy. Whether plasma ice shape regulation could reduce flight risk is a new problem under the wing with continuous ice. The 3D printed ice shapes were arranged on the leading edge of the wing based on NACA0012 airfoil, aiming to simulate the configuration after ice shape regulation. And the aerodynamic parameters were obtained by wind tunnel experiments. The experimental results showed that the ratio of signal regulation ice width $d$ to chord length of the wing $b_{A}$ determined the aerodynamic characteristics, and the aerodynamic characteristics changed better compared with configuration of the continuous ice. However, the flight risk of the wing under given regulation ratio is unknown. Based on the straight and swept wing after regulating, the flight safety boundaries were simulated by the reachable set method. Further, a method of quantitative assessment of flight risk is proposed. Quantitative values of risk were calculated. The results show that the flight risk all decreases from level 2 to level 4 compared with configuration of the continuous ice when $d/b_{A}$ equals 0.15 under conditions of swept and straight wing.

## Introduction

The wing is a key component of the windward side of the aircraft, and ice on the wing will deteriorate the aerodynamic characteristics and deteriorate the flight dynamics^1,2^. Plasma ice shape regulation is a new de-icing technology^3^. Given the actual requirements of airborne de-icing equipment, plasma actuators were arranged in icing sensitive areas, which used thermal effects to cut continuous ice into intermittent ice pieces to achieve de-icing^4^. Plasma de-icing belongs to a kind of heating de-icing system according to de-icing principle. The traditional heating de-icing system includes electric heating de-icing and hot gas de-icing, which has been easy to realize ice tolerance^5,6^. At present, airborne electric heating anti-icing faces technical difficulties such as the development of heating resistor wire, heat conduction and electrical wiring^7^. The hot gas anti-icing system utilizes the engine compressor to induce gas, which has the disadvantages of complicated pipeline arrangement and high energy consumption^8^. These problems limit the development of electric heating de-icing and hot gas de-icing methods to a certain extent. Compared with the common heating de-icing technology, plasma de-icing technology has the advantages of high energy efficiency, fast response, and simple arrangement^9,10^. Ice avoidance is a desirable goal for increased flight safety^11^. Most current ice protection systems target goal is eliminating all the ice at the wing leading edge^12,13^. However, it is not practical with limited energy. Due to energy constraints and space limitations, most UAVs do not equip de-icing systems and can only fly in ice-free weather^14^. The plasma ice shape regulation technology broke the concept of removing all the ice on the wing. And preliminary exploratory plasma ice shape regulation ice wind tunnel tests showed that the regular wavy wing surface alleviated the damage of the aerodynamic performance of the wing with continuous ice. And it is expected to reduce about half power consumption^15,16^. At the same time, the residual ice can be controlled effectively to avoid irregular ice ridges and secondary pollution by planning the layout position and excitation time of the plasma actuator rationally^17^.

The above studies show that plasma ice shape regulation is a de-icing technology with potential for airborne applications. Relying on the regulation of the more dangerous continuous ice to achieve the purpose of ice-tolerant flight, the de-icing effectiveness could not be analyzed by the traditional evaluation method of observing whether or not the ice is removed completely. Previous exploratory ice wind tunnel experiments explored the physical form of plasma regulation of ice shape, and initially showed that the plasma ice shape regulation technique can slow down the deterioration of aerodynamic characteristics^18^. However, the flight risk after plasma ice shape regulation is unknown and it is a new question whether the flight safety can be ensured under ice-tolerant flight conditions. The key problem that restricts the application of plasma ice shape regulation technology is the internal relationship between flight risk and given regulation ratio. A flight risk assessment method is needed to be applied to find the internal relationship. Furthermore, the quantitative assessment of flight risk under different ice shape regulation schemes is the fundamental standard for judging and optimizing this technology. As early as 1929, in order to prevent flight accidents caused by icing, Kopp^19^ and Carroll^20^ have proposed that icing had a more significant impact on flight safety and flight risk, compared with the impact of icing on aircraft weight gain. Calculation of ice tolerant flight and flight safety envelope was a current hot issue. Vukits studied that the best way to avoid natural icing hazards was to have accurate forecasts, know what conditions lead to natural icing, and to avoid them^21^. At present, many scholars tried to construct an effective and accurate method for predicting flight risk under icing condition. A major risk prediction method under icing condition was based on the pilot report, through which pilots conveyed the current icing intensity and estimated whether their aircraft can maintain safe operation before entering the reporting area. There were four intensity levels of icing during flight: trace, light, moderate and severe. However, this definition was subjective, vague and less universal^22,23^. Zeppetelli and Habashi calculated the aerodynamic parameters such as the maximum lift coefficient and drag coefficient of an iced aircraft by Computational Fluid Dynamics (CFD) simulations. Based on these parameters, the flight safety under icing conditions was quantitatively evaluated^24,25^. Professor Bragg reviewed the progress toward developing the technology for a smart icing system. Large icing effects on aircraft have been documented. Good results have been seen using stability and control derivatives and trim values to predict icing level^26,27^. Team of Xu Haojun established coupling dynamics model of icing aircraft aerodynamics and flight dynamics. Based on quantitative assessment and visualization methods of flight risk, the icing risk management system was established, which helped the pilot to realize the possible dangers in advance and perform correct maneuvers^28–30^. And with the Copula theory, the joint distribution model of flight parameters with three distinct distribution types was built. The three-dimensional extreme flight risk probability was defined. Based on the quantitative flight risk, the accident induction mechanism under icing conditions was discussed^31,32^. Mendonça constructed a flight simulation platform and proposed a method of flight safety analysis under icing conditions^33^. Rohit Pandita evaluated dynamic flight envelopes by reachable sets and demonstrated how to evaluate flight safety envelopes at various trim points based on the NASA General Transportation Model (GTM)^34^.

The above scholars have studied the variation of flight risk under icing conditions. The fundamental purpose of plasma ice shape regulation technology aims to reduce flight risk and ensure flight safety. It is feasible to evaluate the effect of plasma ice shape regulation from flight risk. Thus, a more unsolved problem might be “what is the level of flight risk under different regulation schemes and different airfoils?”. Overall, this paper mainly studied: simulating the configurations with continuous ice and regulation ice by 3D Print ice; obtaining the aerodynamic parameters by wind tunnel; calculating the flight safety boundaries based on the reachable set theory under two kinds of wings; establishment of flight risk quantitative index and assessment of flight risk. It provides method guidance for explaining that ice shape regulation can expand flight safety boundary and reduce flight risk. Further, the proposed method provides technical support for formulating the optimal ice shape regulation scheme.

## Aerodynamic parameter acquisition

**Figure 1:**
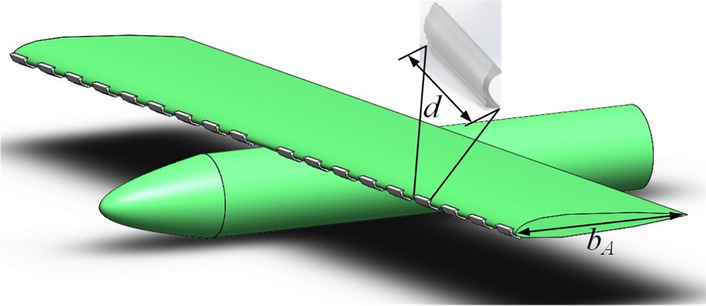
Ice shape regulation diagram based on straight wing.

Based on NACA0012, the previous group conducted plasma ice shape regulation ice wind tunnel experiments and plasma ice shape regulation verification experiment respectively. The former one is a plasma ice shape regulation experiment conducted in an ice wind tunnel. The latter one refers to a wind tunnel based on a scaled wing model with 3D printed ice in the wind tunnel. In the former, the physical morphology of the regulation ice after plasma ice shape regulation was obtained by 3D scan method. The 3D printed ice shape is produced based on 3D scanning of ice shape. Different regulation schemes were designed under the straight wing condition. Further, the group conducted wind tunnel experiments with ice shape regulation and flight experiments with simulated ice at the corresponding regulation ratios. The wind tunnel tests with simulation ice were carried out by research groups as shown in Fig. 1. The regulation ratio $d/b_{A}$ is defined. $d$ is a single regulation ice width. $b_{A}$ is the mean aerodynamic chord of the wing. From the experimental results, it seems that regulation law of the lift coefficient is similar in the flight experiment and the wind tunnel experiment. The validation experiments of plasma ice shape modulation based on a straight wing found that it is more effective in mitigating the hazards to the aerodynamic characteristics under the continuous ice configuration when the regulation ratio is between 0.1 and 0.2. Reynolds number $Re$ in wind tunnel experiments and Reynolds number in flight experiments is 3.70 × 10^5^ and 4.93 × 10^5^–7.40 × 10^5^. It seems that the Reynolds number is not a decisive factor for the ice shape regulation method, but the key is to choose suitable regulation ratio to mitigate the loss of aerodynamic performance under the incoming continuous ice conditions. And the specific experimental data can be found in the reference^15^.

**Figure 2:**
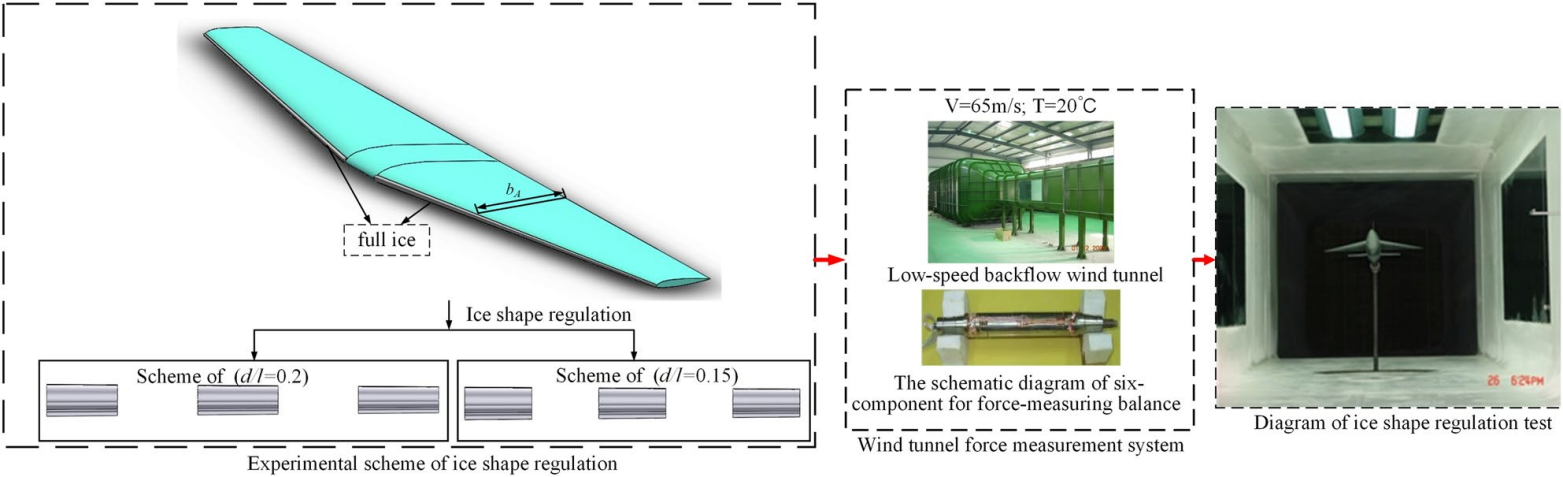
Ice shape regulation experiment based on swept wing.

**Figure 3:**
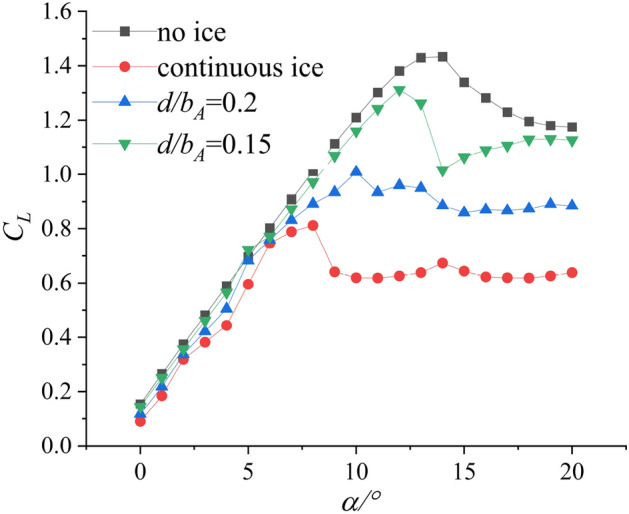
Lift Coefficient $C_{L}$ of background aircraft under four configurations^18^.

**Figure 4:**
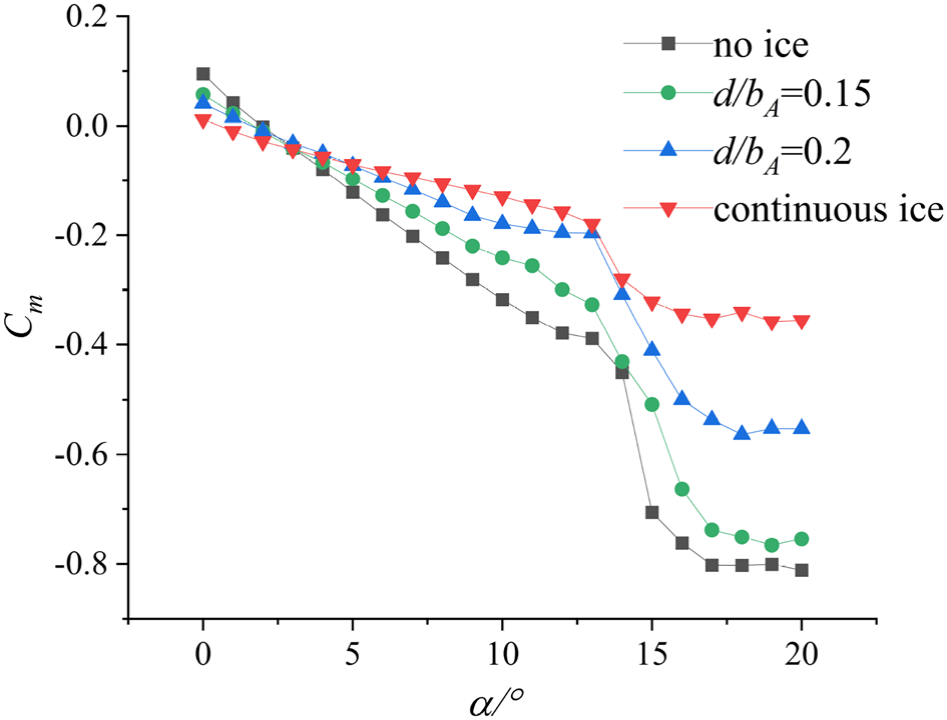
Pitching Moment Coefficient $C_{m}$ of background aircraft under four configurations^18^.

**Figure 5:**
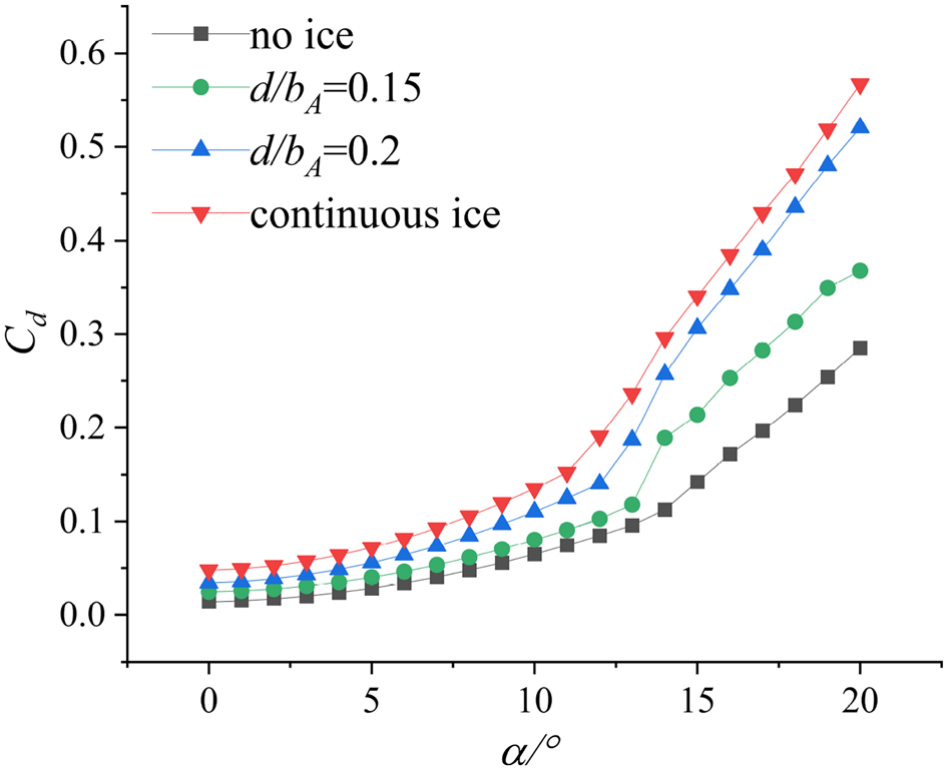
Drag coefficient $C_{d}$ of background aircraft under four configurations^18^.

Reference^18^ conducted a plasma ice shape regulation verification experiment based on the swept wing, which is similar to the straight wing condition. The aerodynamic parameters after ice shape regulation are obtained and compared under different wing conditions. Referring to the experimental results under straight wing conditions, two schemes based on the swept wing are selected. The schemes are ${d \mathord{\left/ {\vphantom {d {b_{A} }}} \right. \kern-0pt} {b_{A} }} = 0.15$ and ${d \mathord{\left/ {\vphantom {d {b_{A} }}} \right. \kern-0pt} {b_{A} }} = 0.2$. As shown in Fig. 2, the incoming flow is 40 m/s and the air temperature is 20 $^\circ C$. The $Re$ of the wing in the wind tunnel test is 3.70 × 10^5^. The range of angle of attack (AOA) is from 0° to 20°. The lift coefficient $C_{L}$, drag coefficient $C_{d}$ and pitching moment coefficient $C_{m}$ were measured under no ice, continuous ice, and schemes of ${d \mathord{\left/ {\vphantom {d {b_{A} }}} \right. \kern-0pt} {b_{A} }} = 0.15$ and ${d \mathord{\left/ {\vphantom {d {b_{A} }}} \right. \kern-0pt} {b_{A} }} = 0.2$ configuration as shown in Figs. 3, 4, 5.

Compared with no ice (black curve) and continuous ice (red curve), drag coefficient increases. Lift coefficient $C_{L}$ and pitching moment coefficient $C_{m}$ decrease under configuration of continuous ice. Compared with configuration of the no ice, the maximum lift coefficient reduces by 53.4% under the continuous ice configuration and the stall (AOA) reduces from 14° to 8° as shown in Fig. 3. The pitch moment coefficient $C_{m}$ changes little in the small AOA region. Taking $\alpha = 13^\circ$ as an example, the pitch moment coefficient reduces by 59.8% under continuous ice configuration as shown in Fig. 4. Compared with the continuous ice (red curve) and schemes of ${d \mathord{\left/ {\vphantom {d {b_{A} }}} \right. \kern-0pt} {b_{A} }} = 0.15$ and ${d \mathord{\left/ {\vphantom {d {b_{A} }}} \right. \kern-0pt} {b_{A} }} = 0.2$ (green and blue curves), the lift coefficient $C_{L}$ and the pitching moment coefficient $C_{m}$ increase. The drag coefficient $C_{d}$ decreases. Compared with the continuous ice and under scheme of ${d \mathord{\left/ {\vphantom {d {b_{A} }}} \right. \kern-0pt} {b_{A} }} = 0.15$ configuration, the maximum lift coefficient $C_{L}$ increases by 38.5% and the drag coefficient $C_{d}$ decreases by 36.1% at stall angle of attack. Under scheme of ${d \mathord{\left/ {\vphantom {d {b_{A} }}} \right. \kern-0pt} {b_{A} }} = 0.15$ configuration, the maximum lift coefficient $C_{L}$ (green curve) increases by 31.4% and the drag coefficient $C_{d}$ decreases by 13.2% at stall AOA as shown in Fig. 5. Meanwhile, the reduction of pitching moment coefficient is 16.1% and 49.2% at $\alpha = 13^\circ$.When the swept wing is selected, the results show that the ice shape regulation technology completes increasing lift and reducing drag under ice conditions. And it is found the improvement effect is better when the scheme of ${d \mathord{\left/ {\vphantom {d {b_{A} }}} \right. \kern-0pt} {b_{A} }} = 0.15$ is selected. The above data can be found in reference^18^. However, the aerodynamic characteristics are not significantly improved under both regulation schemes in the large angle of approach region compared with the no ice condition. Because of the above results, it is unknown whether the ice shape regulation can reduce the flight risk.

## Reachable set theory and calculation of flight safety boundary

The above study obtained the aerodynamic parameters under the condition of swept wing and straight wing. Based on the comparison of aerodynamic parameters between the swept wing and the straight wing, the aerodynamic parameters are obviously changed for the better compared the continuous ice condition with the regulation ratio of 0.15. However, the aerodynamic characteristics under the continuous ice condition are not well improved in the large angle region under several regulation schemes. In this section, the flight safety boundaries of the two wing conditions are simulated based on the reachable set method to provide inputs for the flight risk analysis.

### Reachable set theory and model of aircraft dynamics

Assuming that the nonlinear dynamics of the system are given by Eq. (1).

1 $$ \dot{x} = f\left( {x,t,u} \right) $$

where: $x \in R^{n}$ is the state of the system. $u \in U$ is the input of the system. $t$ is time.

**Figure 6:**
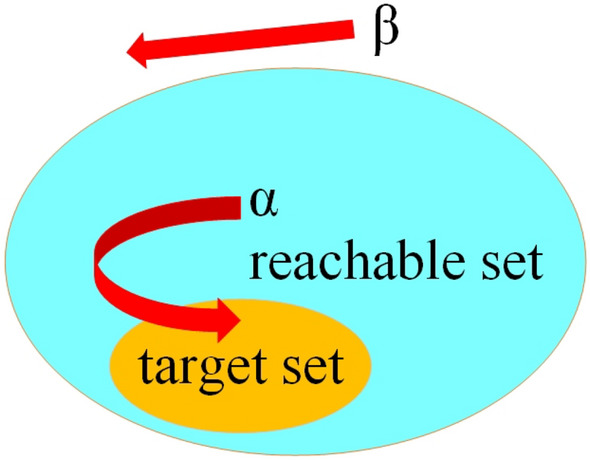
Target set and reachable set.

For the system represented by Eq. (1), the reachable set $P_{\tau } (G_{0} ) \in R^{n}$ and the target set $G_{0} \in R^{n}$ are defined. The reachable set $P_{\tau } (G_{0} ) \in R^{n}$ represents the set of states that can enter the target set $G_{0} \in R^{n}$ at time $t \in \left[ {0,\tau } \right]$ under the action of the input variable $u \in U$. The dynamics of the system can evolve backward and forward in time, producing backward and forward reachable sets^35,36^. For a forward reachable set, initial conditions are specified, and the set of all states that can be reached along the trajectory starting in the initial set is determined. For the backward reachable set, a set of target states is defined, and a set of starting states that can reach the target set is determined. For example, the $\alpha$ point is a point in the reachable set, which can be returned to the target set by control. The $\beta$ point is a point outside the reachable set and cannot return to the target set in Fig. 6. Different forms of reachable sets have different focuses, among which backward reachable sets can be used to recover from unexpected states. Icing aircraft has a high degree of uncertainty and flight risk. In this paper, the reachable set method is introduced into the inscription of flight safety boundary under icing conditions.

The reachable set can be calculated by the level set method. The level set method is a numerical algorithm used for specific types of partial differential equations. It is widely used in the tracking of dynamic boundaries. The boundary of the system is determined by solving the time-dependent Hamilton Jacobin partial differential equations.

2 $$ \frac{{\partial \phi \left( {x,t} \right)}}{\partial t} + H\left( {x,p,u} \right) = 0,\,\,\phi \left( {x,T} \right) = C, $$

where,

3 $$ H\left( {x,p,u} \right) = \mathop {{\text{sup}}}\limits_{u \in U} p^{T} \cdot f\left( {x,u} \right). $$

Here,

4 $$ p = \frac{{\partial \phi \left( {x,t} \right)}}{\partial x} = \nabla \phi \left( x \right). $$

The optimal control input is:

5 $$ u^{*} \left( {x,p} \right) = \arg \max p^{T} f\left( {x,t,u} \right) $$

In the process of calculating the boundary of the system, $x$ is the state of the system, and $p$ is the change gradient of $\phi \left( {x,t} \right)$, which represents the expansion direction of the boundary. Adjust the system state equation by applying control input $u$ to make $H\left( {x,p,u} \right)$ in Eq. (3) take the extreme value. In this way, the implicit function of the boundary of $\phi \left( {x,t} \right)$ in Eq. (2) changes at the fastest speed, making the boundary of the system within a finite time the largest. If the time is long enough when the boundary change gradient $p$ is perpendicular to the system state, that is, $H\left( {x,p,u} \right)$ is equal to zero, and the derivative of the boundary implicit function $\phi \left( {x,t} \right)$ with respect to $t$ is equal to zero. At this time, the boundary no longer changes, that is, the boundary of the system is obtained.

The introduction of reachable set theory, as well as the solution process and step analysis of reachable set can be referred to reference^37^.

It is necessary to calculate the controllable range of altitude, speed, and flight path angle when the flight safety boundaries are calculated. The longitudinal dynamics model based on a model aircraft is established as shown in Eqs. (6), (7), (8), (9)^18^:

6 $$ \dot{V} = \frac{1}{m}\left( {T\cos \alpha - D - mg\sin \gamma } \right) $$

7 $$ \dot{\gamma } = \frac{1}{mV}\left( {T\sin \alpha + L - mg\cos \gamma } \right) $$

8 $$ \dot{q} = \frac{{QS_{ref} \overline{c}C_{m} }}{{J_{y} }} $$

9 $$ \dot{\alpha } = q - \dot{\gamma }. $$

Here:

10 $$ L = \frac{1}{2}\rho S_{ref} V^{2} C_{L} $$

11 $$ D = \frac{1}{2}\rho S_{ref} V^{2} C_{D} $$

12 $$ M = \frac{1}{2}\rho S_{ref} V^{2} C_{m} $$

where: $m$ is the quality of aircraft. $g$ is the acceleration of gravity. $\rho$ is atmospheric density. $V$ is the flight speed. $\dot{V}$ is derivative of velocity. $\dot{\gamma }$ is the derivative of flight path angle. $\dot{H}$ is the derivative of height $\gamma$ is the flight path angle. $q$ is the pitch angle speed. $\alpha$ is the angle of attack. $\overline{c}$ is the mean aerodynamic chord of the wing. $J_{y}$ is the rotational inertia of the aircraft to the $y$ axis.$D$ is the drag of aircraft. $T$ is the thrust of aircraft. $L$ is the lift of aircraft. $M$ is pitching moment of aircraft. $S_{ref}$ is the area of wing. $C_{m}$ is the pitching moment coefficient. $C_{L}$ is lift coefficient. $C_{D}$ is drag coefficient in Eqs. (10), (11), (12)^38^.

### Calculation of flight safety boundary

#### Swept wing condition

Aircraft landing phase is selected as the case. The aircraft is prone to ice during the landing phase, threatening flight safety. In severe cases, the aircraft is uncontrollable, caused by flight accidents. And the landing phase flight speed $V$, flight pitch angle $\gamma$, pitch angle speed $q$ and other parameters need to be strictly restricted. The angle of attack $\alpha$ and aircraft thrust $T$ is regarded as the input of the system, and the value range is $\alpha \in \left[ {0,20^\circ } \right]$, $T \in \left[ {10,200N} \right]$. We choose the three performance indicators of flight speed $V$, flight path angle $\gamma$, and pitch angle speed $q$ as the output to determine the flight safety boundary. Three state parameters of $V$, $\gamma$, and $q$ are selected to construct three-dimensional state space. $\left[ {V_{min,} V_{max} } \right] = [10,56][10,56]$, $\left[ {\gamma_{min,} \gamma_{max} } \right] = [0.52,0.69]$ and $\left[ {q_{min,} q_{max} } \right] = [0.87,1.22]$ are the constraint range of three state parameters at a certain moment, which can form a three-dimensional state space. The state space is regarded as target set. The errors in the characterization results of reachable sets are mainly determined by the number of computational grids. The more the number of grids is gotten and the higher the calculation accuracy has. But the calculation time will become longer. To shorten the calculation time and ignore the calculation accuracy error, the number of grids should be selected appropriately during calculation. The number of grids selected in the three directions of flight speed, flight pitch angle and pitch angle speed are 72, 70, 72. The reachable set is extended from the target set, which is safe in the reachable set during landing phase. If the aircraft deviates from the reachable set, the state parameters may not return to the target set. The pilot will not manipulate the aircraft back to safe states, and the landing phase will enter a dangerous state. The reachable set can be regarded as the flight safety boundary of the aircraft. According to the aerodynamic parameters obtained in the second section, the safety boundaries under no ice, continuous ice, and two kinds of regulation ice shape configurations are calculated.

**Figure 7:**
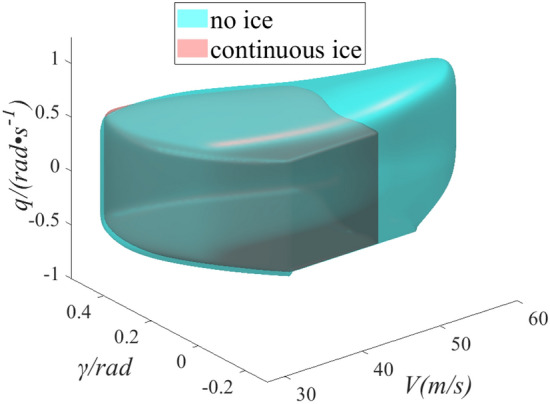
Comparison of flight safety boundary between no ice and continuous ice.

As shown in Fig. 7, the range of reachable set can be regard as the range of flight safety boundary. When the state parameters are within the safe boundary, flight safety can be guaranteed. And it can ensure a safe landing, when an external disturbance or pilot maneuver is within the safe boundary. The aerodynamic characteristics of the aircraft become worse after icing, so the flight safety boundary between the no ice and continuous ice configurations is significantly different. Blue surface is the flight safe boundary under no ice configuration and pink is under continuous ice configuration. Compared with no ice configuration, the safety boundary shrinks under the continuous ice. The volume of safety boundary reduces by 25%, which causes the reduction of safety margin. The scope of the pilot can safely operate is limited, so the pilot needs to be careful driving. If the pilot does not realize the reduction of safe operating range under icing, slightly will lead to flight accidents.

**Figure 8:**
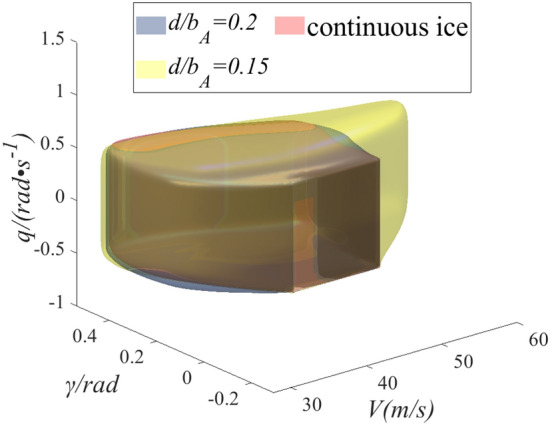
Comparison of flight safety boundary between regulation ice shape and continuous ice.

As shown in Fig. 8, the flight safety boundary between continuous ice and two schemes of ${d \mathord{\left/ {\vphantom {d {b_{A} }}} \right. \kern-0pt} {b_{A} }} = 0.15$ and ${d \mathord{\left/ {\vphantom {d {b_{A} }}} \right. \kern-0pt} {b_{A} }} = 0.2$ are compared. The yellow and gray surfaces are flight safety boundaries under schemes of ${d \mathord{\left/ {\vphantom {d {b_{A} }}} \right. \kern-0pt} {b_{A} }} = 0.15$ and ${d \mathord{\left/ {\vphantom {d {b_{A} }}} \right. \kern-0pt} {b_{A} }} = 0.2$ configurations. Compared with the continuous ice configuration, the safety boundary has been expanded under the two kinds of regulation ice. The safety boundary configuration has been expanded by 10% under scheme of ${d \mathord{\left/ {\vphantom {d {b_{A} }}} \right. \kern-0pt} {b_{A} }} = 0.15$ and scheme of ${d \mathord{\left/ {\vphantom {d {b_{A} }}} \right. \kern-0pt} {b_{A} }} = 0.2$ has been expanded by 5%. In the direction of the flight pitch angle, the range of safe control is increased. Mainly because the regulation ice state slows down the damage of the flow field around the wing under icing and further slows down the early separation of the airflow. The range of safe control is obviously increased in the speed direction. For pilots, the uncontrollable factors reduce and the flight risk slows down.

**Figure 9:**
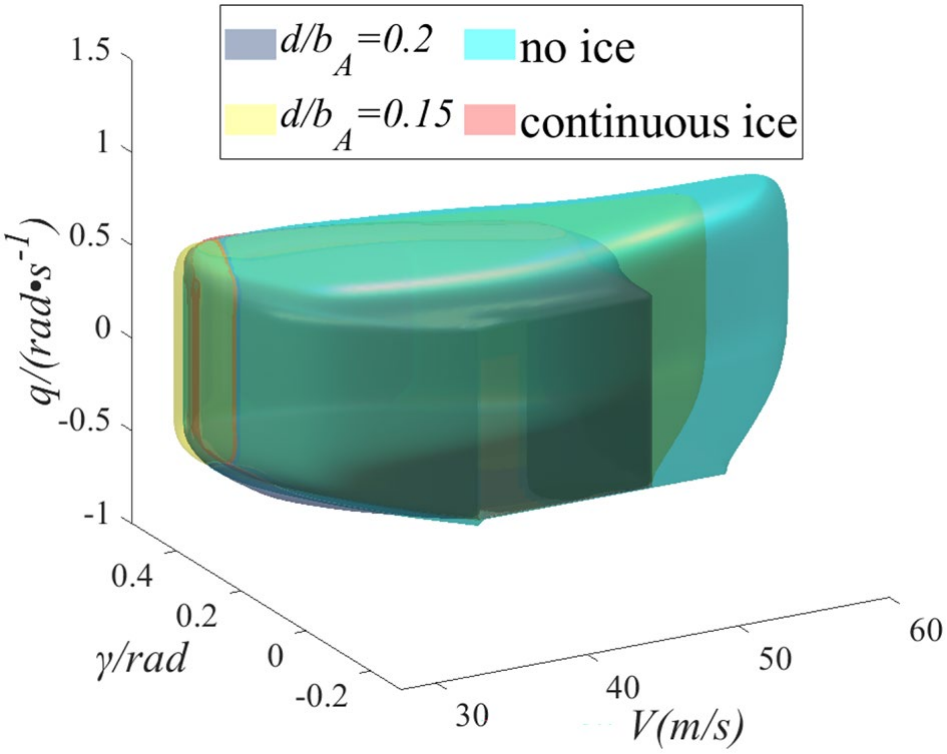
Comparison of flight safety boundary under four sates.

As shown in Fig. 9, the flight safety boundaries are compared under the four configurations. The velocity direction and the pitch angular velocity direction shrink obviously under continuous ice state. Icing causes deterioration of the lift and drag characteristics. Further, the pilot will pull the rod to increase the AOA, which will easily cause the flight pitch angle and pitch angle rate to exceed the specified range. When the same thrust is input, the flight speed is significantly lower than no ice and regulation ice in the speed direction because of the increase of drag under icing. Compared with continuous ice state, flight safety boundary also increases under regulation ice configuration in pitch angle velocity direction. The state parameters have larger range in the pitch direction. The pilot can manipulate to change the dangerous state, ensuring a safe landing. In conclusion, the flight safety boundary under icing has been expanded by ice shape regulation. Especially, the flight safety boundary expansion is more obvious under scheme of ${d \mathord{\left/ {\vphantom {d {b_{A} }}} \right. \kern-0pt} {b_{A} }} = 0.15$ configuration, and the flight safety is enhanced.

**Figure 10:**
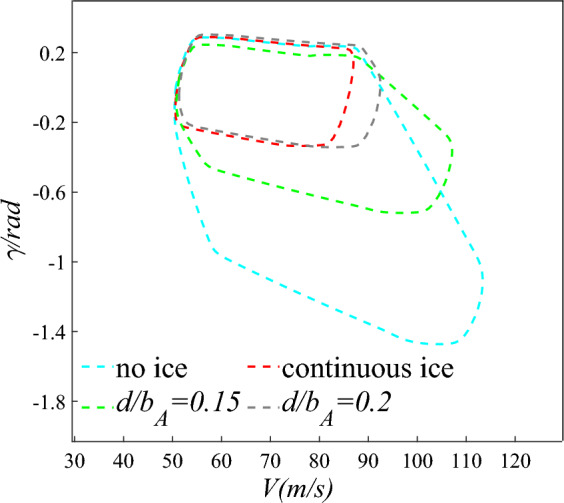
Two-dimensional safety boundary diagram under four configurations.

The above results found that the aircraft state parameters shrink more obvious in the direction of flight speed $V$ and flight pitch angle $\gamma$ under icing. As shown in Fig. 10, the two-dimensional safety boundary is further characterized by $V$, $\gamma$. $\left[ {V_{min,} V_{max} } \right] = [60,85]$, $\left[ {\gamma_{min,} \gamma_{max} } \right] = [ - 0.35,0.35]$ are the constraint range of two state parameters at a certain moment. The number of grids selected in the three directions of flight speed and pitch angle speed are 500 and 500.

Where: Blue curve is the flight safety boundary under no ice configuration. Red is continuous ice. Green is scheme of ${d \mathord{\left/ {\vphantom {d {b_{A} }}} \right. \kern-0pt} {b_{A} }} = 0.15$. Gray is scheme of ${d \mathord{\left/ {\vphantom {d {b_{A} }}} \right. \kern-0pt} {b_{A} }} = 0.2$. As shown in Fig. 10, the two-dimensional safety boundaries of the four configurations are compared. Compared with the no ice configuration, flight safety boundary reduces by 70.1% under continuous ice configuration. The safety boundary shrinks obviously in the direction of flight speed and flight pitch angle. And the probability of risk events will increase sharply. Compared with the continuous ice configuration, the flight safety boundary increases by 1.17 and 0.32 times under scheme of ${d \mathord{\left/ {\vphantom {d {b_{A} }}} \right. \kern-0pt} {b_{A} }} = 0.15$ and scheme of ${d \mathord{\left/ {\vphantom {d {b_{A} }}} \right. \kern-0pt} {b_{A} }} = 0.2$. Compared with the continuous ice configuration, the flight safety domain has been expanded, but it has not yet reached the no ice configuration.

#### Straight wing condition

**Figure 11:**
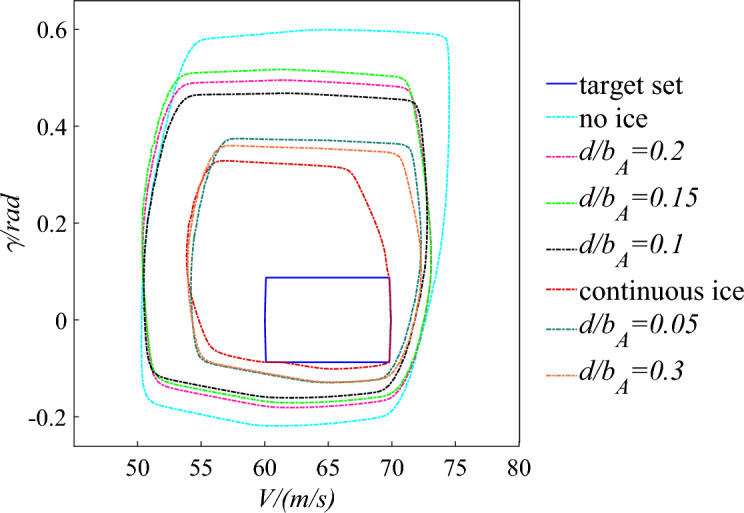
Flight safety boundary under straight wing condition.

The wind tunnel tests with schemes of ${d \mathord{\left/ {\vphantom {d {b_{A} }}} \right. \kern-0pt} {b_{A} }} = 0.05$, ${d \mathord{\left/ {\vphantom {d {b_{A} }}} \right. \kern-0pt} {b_{A} }} = 0.1$, ${d \mathord{\left/ {\vphantom {d {b_{A} }}} \right. \kern-0pt} {b_{A} }} = 0.15$, ${d \mathord{\left/ {\vphantom {d {b_{A} }}} \right. \kern-0pt} {b_{A} }} = 0.2$, and ${d \mathord{\left/ {\vphantom {d {b_{A} }}} \right. \kern-0pt} {b_{A} }} = 0.3$ were carried out in reference^15^. The results show that the better lift coefficient is obtained when schemes of ${d \mathord{\left/ {\vphantom {d {b_{A} }}} \right. \kern-0pt} {b_{A} }} = 0.1 - 0.2$ are selected under straight wing condition. And the improvement effect is the most obvious when scheme of ${d \mathord{\left/ {\vphantom {d {b_{A} }}} \right. \kern-0pt} {b_{A} }} = 0.15$ is selected. Furthermore, the two-dimensional safety boundary is calculated based on the aerodynamic data in reference^15^ in the same flight phase. And the safety boundary is characterized with the performance parameters of velocity $V$ and flight pith angle $\gamma$ as shown in Fig. 11.

Compared with the no ice configuration, the flight safety boundary reduces by 50% under the continuous ice configuration. In the velocity direction, the flight speed is smaller when the engine inputs the same thrust. The main reason is that the drag increases and the lift decrease under icing condition. On the other hand, the boundary also significantly reduces in the flight pith angle direction. That is because that the stall AOA decreases under icing conditions and the limited range of AOA becomes smaller. Through the ice shape regulation, the flight safety boundary is obviously larger compared with the continuous ice configuration when schemes of ${d \mathord{\left/ {\vphantom {d {b_{A} }}} \right. \kern-0pt} {b_{A} }} = 0.1 - 0.2$ are selected. Especially when schemes of ${d \mathord{\left/ {\vphantom {d {b_{A} }}} \right. \kern-0pt} {b_{A} }} = 0.15$ is selected, the flight safety boundary expands by 62.5%. Although the safety boundary does not significantly expand, it expands by 26.6% and 20% compared with the continuous ice configuration when the schemes of ${d \mathord{\left/ {\vphantom {d {b_{A} }}} \right. \kern-0pt} {b_{A} }} = 0.05$ and ${d \mathord{\left/ {\vphantom {d {b_{A} }}} \right. \kern-0pt} {b_{A} }} = 0.3$ are selected. It can be seen that can the flight safety boundary is effectively expanded under icing condition by applying rational arrangement of plasma actuators. Flight risk reduce when residual ice still exists after de-icing.

## Process and calculation of flight risk quantification

### Quantitative assessment parameters of flight risk

**Figure 12:**
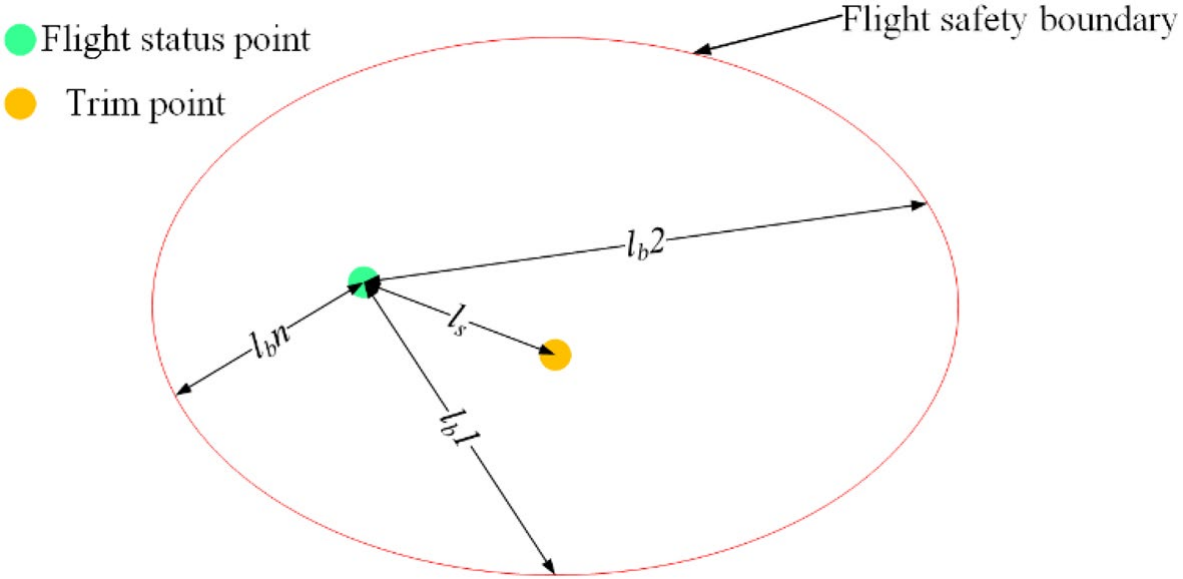
Diagram of quantitative parameter.

The above results show that the flight status is within the flight safety boundary and the aircraft will have risk events rarely due to instability. But the flight status is outside the boundary and risk events will occur. Therefore, the distance between the flight status point and the flight safety boundary is used to quantify the flight risk during the aircraft landing phase. The range of status parameters will be limited by flight control system according to the original control law during flight, so as to reduce the flight risk. Due to the uncertain coupling relationship between flight status under icing conditions, the probability of failure of the original control law will increase. When the flight status is close to the boundary, the state parameters are vulnerable to exceed the safety boundary under external disturbances. Therefore, the distance between the flight status point and the flight safety boundary is regard as the risk quantification index $FR$ at a certain time, as shown in Eq. (13).

13 $$ FR = 1 - \frac{{l_{b} }}{{l_{s} }} $$

where: $FR$ (flight risk) is the quantitative value of flight risk. $l_{b}$ is the nearest distance $Min_{{l_{b} }}$ between the aircraft flight status point and the safety boundary. $l_{s}$ is the distance between flight status point to trim point, as shown in Fig. 12.

According to the position of the trim point, the distance between the flight status point and the trim point can be expressed as Eq. (14).

14 $$ l_{s} = \sqrt {\left( {x_{0} - a_{0} } \right)^{2} + \left( {y_{0} - b_{0} } \right)^{2} } $$

where: $x_{0}$ and $y_{0}$ represent the coordinate value of the flight status point at this time. $a_{0}$ and $b_{0}$ represent the coordinate value of the trim point.

According to Eq. (14), $l_{b}$ can be expressed as Eq. (15).

15 $$ l_{b} = Min\left\{ {\sqrt {\left( {x_{i} - x_{0} } \right)^{2} + \left( {y_{i} - y_{0} } \right)^{2} } } \right\} = Min\left\{ {l_{b} 1,l_{b} 2, \ldots l_{b} n} \right\} $$

where: $x_{i}$ and $y_{i}$ represent the coordinate value corresponding to each point on the security boundary.

According to Eq. (15), when the flight status point is within the safety boundary but close to the boundary, a warning should be given to reserve enough safe steering range for the control system and the pilot. Therefore, the flight risk level $R_{d}$ is classified according to Eq. (16).

16 $$ R_{d} = \left\{ {\begin{array}{*{20}c} {4 \, FR \in \left( {0.2,\left. 1 \right]} \right.} \\ {3 \, FR \in \left( 0 \right.,\left. {0.2} \right]} \\ {2 \, FR \in \left( { - 1,\left. 0 \right]} \right.} \\ {1 \, FR \in \left( { - \infty ,\left. 1 \right]} \right.} \\ \end{array} } \right. $$

**Figure 13:**
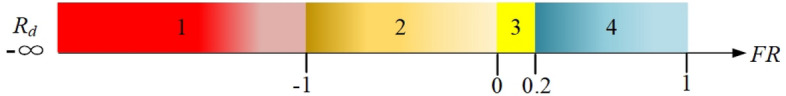
Visualization of flight risk level.

As shown in Eq. (16), $R_{d}$ is the flight risk level. The airplane can be considered to be in a safe state. From the point of view of the reachable set, the flight parameter points are in the reachable set at this time, and can be accessed as in the target set when $R_{d}$ equals 4. When $R_{d}$ equals 3, flight status point is within the flight safety boundary. But flight status point is close to the boundary. At this moment, the flight control system should issue an alarm signal. The probability of risk events increases. When $R_{d}$ equals 2 and 1, the flight status is outside the safety boundary but close to the boundary. Where, the flight parameters are outside the safety boundary but closer to it when $R_{d}$ equals 2. Aircraft maybe come back to safety status by right manipulation of pilots or the correct instructions of the flight control system. When $R_{d}$ equals 1, The flight status is outside the safety boundary and far from the boundary. The aircraft has entered an extremely dangerous state at this time, so that the flight status is not easy to change. From the point of view of the reachable set, the flight state parameters are far away from the region where the reachable set is located, and after controlling them, they still cannot return to the target set. Risk events will be prone to take place. As shown in Fig. 13, to more clearly compare the flight risk of the flight state point when the external conditions change, a flight risk ribbon diagram is constructed to visualize the risk level.

### Flight risk quantification process

**Figure 14:**
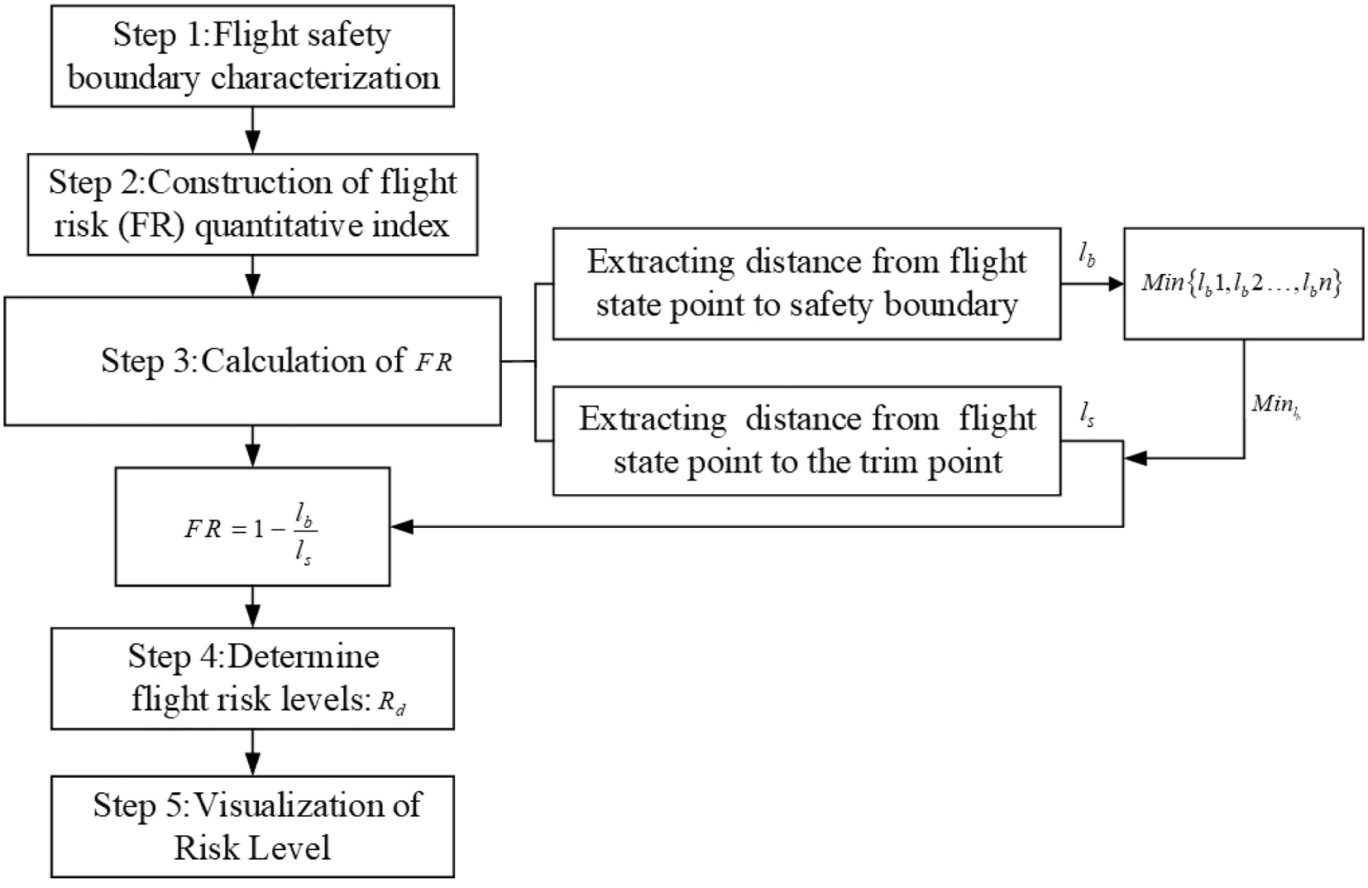
Process of Flight Risk Quantitative Assessment.

The above research mainly evaluated different ice shape regulation schemes under straight wing and swept wing conditions based on aerodynamic characteristics. It is found that better aerodynamic performance is obtained when ${d \mathord{\left/ {\vphantom {d {b_{A} }}} \right. \kern-0pt} {b_{A} }} = 0.1{ - }0.2$ under straight wing condition. Comparing two schemes of ${d \mathord{\left/ {\vphantom {d {b_{A} }}} \right. \kern-0pt} {b_{A} }} = 0.15$ and ${d \mathord{\left/ {\vphantom {d {b_{A} }}} \right. \kern-0pt} {b_{A} }} = 0.2$, it is found that the aerodynamic performance is better when ${d \mathord{\left/ {\vphantom {d {b_{A} }}} \right. \kern-0pt} {b_{A} }} = 0.15$ under swept wing condition. However, the purpose of applying the de-icing method aims to reduce flight risk under icing condition. The flight risk accident is a small probability event. Once it occurs, it will bring serious harm. This part studies the flight risk under residual ice condition after applying ice shape regulation method. As shown in Fig. 14, the reachable set theory is introduced into the characterization of flight safety boundaries, and the reachable set represents the safety boundary under different situations. Then, the nearest distance between the flight state point and boundary $Minl_{b}$ is regard as the quantitative parameter. Furthermore, the flight risk (FR) is constructed to calculate the flight risk level under different situations. Flight risk level is visualized under different regulation schemes and different wings.

### Calculation results and analysis

This part quantifies the flight risk based on the two-dimensional reachable set without considering the influence of pitch angle rate through the above flight risk quantification process. The schemes of ice shape regulation under swept and straight airfoil condition are evaluated from the perspective of flight risk.

#### Swept wing condition

**Figure 15:**
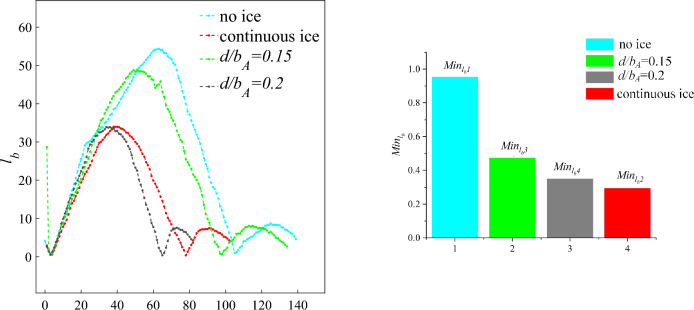
Distance between flight status point and safety boundary under four configurations.

Based on the above method of risk quantification, the level of flight risk is calculated under four configurations including no ice, continuous ice and scheme of in landing phase. The flight status is set to $H = 600\,m,V = 58\,m/s$ at this time. According to the aerodynamic parameters obtained in Sect. "Aerodynamic parameter acquisition", the aircrafts are trimmed under four configurations including no ice, continuous ice, and schemes of $d/b_{A} = 0.2$, $d/b_{A} = 0.15$. As shown in Fig. 15, the values of the distance $l_{b}$ between the flight status point and the safety boundary are calculated based Eq. (15) under four configurations. As shown in Table 1, the values of the distance $l_{s}$ between the flight status point and the trim point are calculated based Eq. (14) under four configurations.

**Table 1:** Calculation of $FR$ under four configurations.

Icing condition	$l_{s}$	$l_{b}$	$FR$	Flight risk level
No ice	1.2707	0.9530	0.2495	Level 4
Continuous ice	0.7276	0.4730	0.3499	Level 4
$d/b_{A} = 0.2$	0.3884	0.3502	0.0984	Level 3
$d/b_{A} = 0.15$	0.2430	0.2941	-0.2102	Level 2

**Figure 16:**
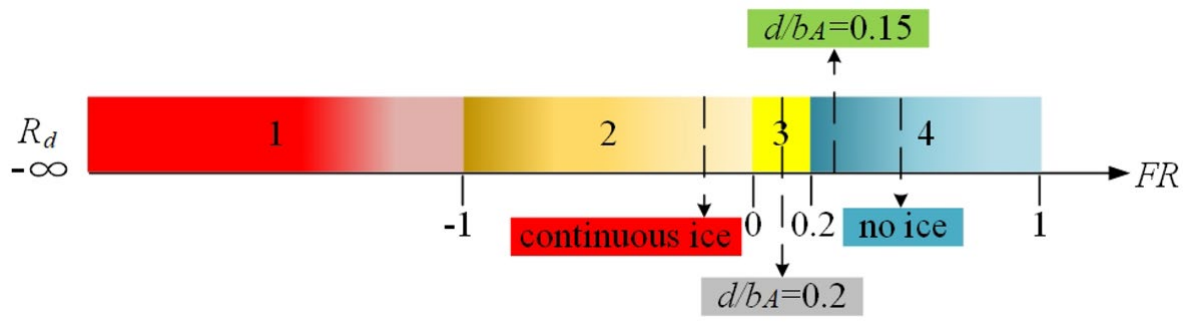
Visualization flight risk level under four configurations.

As shown in Fig. 16, the flight risk levels under the four configurations are marked on the ribbon diagram to visualize the flight risk levels. The results show that the ribbon diagram is blue under no ice state. The flight risk is level 4 and the aircraft is in safe state. The ribbon diagram becomes orange under continuous configuration. The flight risk is upgraded to level 2 and the flight state is already outside the flight safety boundary. At this time, the flight status is gradually losing stability with a high flight risk, which is easy to cause risk events. Ribbon diagram becomes lighter and gradually away from the dangerous state by the ice shape regulation. The flight risk level is reduced. Compared with the two ice regulation schemes, the scheme of $d/b_{A} = 0.2$ is selected and the ribbon diagram changes from orange to yellow. The flight risk level is reduced to level 2, which indicates that the flight state is very close to the safety boundary. According to the above method of risk assessment, warning signals are issued to alert pilots of impending risk events. The scheme of $d/b_{A} = 0.15$ is selected and the ribbon diagram changes from yellow to blue. The flight risk reduces to level 4, and the aircraft is out of danger. However, compared with the no ice configuration, the risk quantification parameters are smaller. It indicates that the existence of partial ice can be allowed under the premise of ensuring flight safety by rationally arranging plasma actuators.

#### Straight wing condition

**Figure 17:**
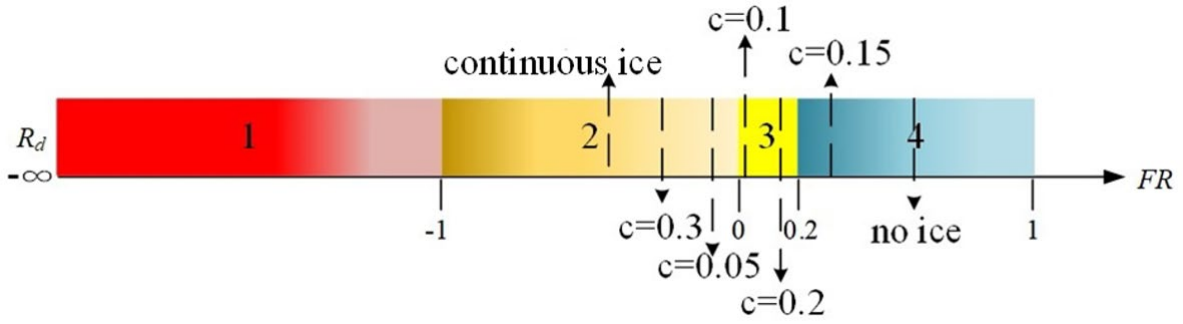
Visualization flight risk level under straight wing condition.

Flight risk level under swept wing condition is analyzed in Sect. “Swept wing condition”. When the straight wing is selected, the risk quantification values of different ice shape regulation schemes are calculated under the same flight state by the risk quantification method in Sect. "Aerodynamic parameter acquisition". The calculation results are shown in the Table 2. And the risk value is visualized, as shown in the Fig. 17.

**Table 2:** Calculation of $FR$.

Icing condition	$l_{s}$	$l_{b}$	$FR$	Flight risk level
No ice	0.3058	0.5771	0.47	Level 4
Continuous ice	0.1983	0.1239	− 0.6	Level 2
$d/b_{A} = 0.05$	0.2207	0.1698	− 0.3	Level 2
$d/b_{A} = 0.15$	0.2539	0.2987	0.15	Level 4
$d/b_{A} = 0.1$	0.2909	0.4215	0.31	Level 3
$d/b_{A} = 0.2$	0.2428	0.2555	0.05	Level 3
$d/b_{A} = 0.3$	0.2218	0.1848	− 0.2	Level 2

The flight risk level is level 4 under the no ice configuration, which indicates that the aircraft is within the flight safety boundary and far away from the flight safety boundary. And it is not easy to deviate from the stable state with exterior disturbances. The flight risk increases to level 2 under continuous ice configuration. At this time, the aircraft is outside the flight safety boundary. The main reason is that the aerodynamic characteristics are seriously deteriorated under icing condition and the stability characteristics seriously affected. The probability of dangerous events increases. The color of the ribbon image changes from the attention level alarm to the consulting level alarm by the ice shape regulation, indicating that the flight safety boundary is expanded at this time. The aircraft is still outside the boundary but close to the boundary when the schemes of $d/b_{A} = 0.05$ and $d/b_{A} = 0.3$ are selected. At this time, the pilot can change the flight state back to the stable state by manipulating the aircraft correctly.

The flight risk obviously reduces when the schemes of $d/b_{A} = 0.1 - 0.2$ are selected. The aircraft is within the safety boundary but still close to the safety boundary when the schemes of $d/b_{A} = 0.1$ and $d/b_{A} = 0.2$ are selected. The pilot needs to control the aircraft carefully. The main reason is that flight state parameters are easy to exceed the limit, which is easy to cause the aircraft to enter the dangerous state and make the flight state difficult to change. The aircraft is within the boundary and far away from the boundary the schemes of $d/b_{A} = 0.15$. The range that pilots manipulate the aircraft increases and the probability of dangerous events decreases.

In conclusion, the flight risk assessment is compared under ice shape regulation condition when different airfoils are selected. The results show that the flight risk under continuous ice condition greatly reduces. Meanwhile, flight risk significantly reduces under continuous ice configuration when schemes of $d/b_{A} = 0.1 - 0.2$ are selected under straight wing condition and scheme of $d/b_{A} = 0.15$ is selected under swept condition.

## Conclusions

The above research studies the flight risk quantification method based on the reachable set theory, and evaluates the flight risk under two kinds of airfoils. The flight quality was evaluated based on small disturbance equation. The results show:

1. The wind tunnel test results based on swept wing show that the maximum lift coefficient increases by 38.5% and the drag coefficient decreases by 36.1% when scheme of $d/b_{A} = 0.15$ is selected. It is consistent with test results under straight wing conditions. The aerodynamic characteristics is less degradation compared with the condition of continuous ice.
2. The safety boundaries are calculated under two kinds of wings. The results show that the safety boundary is diminished under continuous ice conditions. Through ice shape regulation, the safety boundary does not reach no ice configuration. But compared with the continuous ice configuration, the safety boundary is all expanded. Among them, the two-dimensional safety boundary is extended by 117% and 62.5% under swept and straight wing when the scheme of $d/b_{A} = 0.15$ is selected.
3. A flight risk assessment method is proposed based on reachable set theory. The flight risk under two wings was evaluated. The risk level was raised from level 2 to level 4 under straight and swept wing condition when scheme of $d/b_{A} = 0.15$ is selected.

In summary, the flight risk under icing condition is better decreased and the flight quality is better improved under the scheme of $d/b_{A} = 0.15$ condition. Flight safety manipulation space is extended, increasing margin of controllability for pilots. More detailed research will be conducted to evaluate the effect of ice shape regulation schemes under different ice shape. In addition, the proposed method is used to find the optimal ice shape regulation scheme and provides optimization method reference for other electric heating de-icing methods.

## Data Availability

All data generated or analyzed during this study are included in this published article and the datasets used during the current study available from the corresponding author on reasonable request.
